# Asperflavin, an Anti-Inflammatory Compound Produced by a Marine-Derived Fungus, *Eurotium amstelodami*

**DOI:** 10.3390/molecules22111823

**Published:** 2017-10-29

**Authors:** Xiudong Yang, Min-Cheol Kang, Yong Li, Eun-A. Kim, Sung-Myung Kang, You-Jin Jeon

**Affiliations:** 1 College of Chemical and Pharmaceutical Engineering, Jilin Institute of Chemical Technology, Jilin 132022, China; yangwt_1981@163.com; 2 Department of Marine Life Science, Jeju National University, Jeju 690-756, Korea; networksun@naver.com (M.-C.K.); yellow6798@hanmail.net (E.-A.K.); 3 College of Pharmacy Science, Changchun University of Traditional Chinese Medicine, Changchun 130017, China; donaikun@163.com; 4 Pediatric Oncology Experimental Therapeutics Investigators Consortium (POETIC) Laboratory for Pre-Clinical and Drug Discovery Studies, University of Calgary, Calgary, AB T2N1N4, Canada; tjdaud81@gmail.com; 5 Marine and Environmental Research Institute, Jeju National University, Jeju 695-814, Korea

**Keywords:** asperflavin, marine fungus, Anti-inflammation, *Eurotium amstelodami*, NO production

## Abstract

In the present study, 16 marine-derived fungi were isolated from four types of marine materials including float, algae, animals and drift woods along with the coast of Jeju Island, Korea and evaluated for anti-inflammatory effects in lipopolysaccharide (LPS)-stimulated RAW 24.7 cells. The broth and mycelium extracts from the 16 fungi were prepared and the broth extract (BE) of *Eurotium amstelodami* (015-2) inhibited nitric oxide (NO) production in LPS-stimulated RAW 264.7 cells without cytotoxicity. By further bioassay-guided isolation, three compounds including asperflavin, neoechinulin A and preechinulin were successfully isolated from the BE of *E. amstelodami*. It was revealed that asperflavin showed no cytotoxicity up to 200 μM and significantly inhibited LPS-induced NO and PGE2 production in a dose-dependent manner. In the western blot results, asperflavin suppressed only inducible NOS (iNOS), but COX-2 were slightly down-regulated. Asperflavin was also observed to inhibit the production of pro-inflammatory cytokines including TNF-α, IL-1β, and IL-6. In conclusion, this study reports a potential use of asperflavin isolated from a marine fungus, *E. amstelodami* as an anti-inflammatory agent via suppression of iNOS and pro-inflammatory cytokines as well as no cytotoxicity.

## 1. Introduction

As marine microorganisms have proved their value as sources of bioactive compounds, they have attracted more attention due to their diverse living environment. In the past few decades, marine-derived fungi have been proven to be an important source of novel and bioactive natural products. So far, thousands of secondary metabolites from marine-derived fungi have been reported [1]. Metabolites with unique structural features have been found in marine fungi that greatly differ from those found in terrestrial counterparts. Furthermore, most of these compounds were found to possess antioxidant, antimicrobial, anti-cancer and anti-inflammatory effects in vitro or in vivo [2]. The majority of the isolated compounds (almost 50%) belonged to polyketides and their isoprene hybrids, followed by alkaloids, terpenes, and peptides, which contributed 14–20%. These new compounds were produced mostly from members of the fungal genera *Penicillium* and *Aspergillus*. Representatives of other genera including *Acremonium*, *Emericella*, *Epicoccum*, *Exophiala*, *Paraphaeospaeria*, *Phomopsis*, and *Halarosellinia* were less common [3].

Inflammation is a beneficial host response to foreign challenge or tissue injury that ultimately leads to the restoration of tissue structure and function [4]. However, prolonged inflammation can be harmful, leading to the pathogenesis of many diseases. Macrophages play an important role in host defenses against noxious substances and are involved in a variety of disease processes including autoimmune diseases, inflammatory disorders and infections [5]. They are potent secretory cells that release an array of mediators, including pro-inflammatory and cytotoxic cytokines as well as growth factors, bioactive lipids, hydrolytic enzymes, reactive oxygen intermediates, and nitric oxide (NO), all of which have been implicated in the pathogenesis of tissue injury [6,7]. Macrophages are activated by IFN-γ, pro-inflammatory cytokines and lipopolysaccharide (LPS). Activated macrophages play an important role in inflammatory diseases via production of cytokines, including interleukin-1β (IL-1β), tumor necrosis factor-alpha (TNF-α), and IL-6 [8,9,10]. These inflammatory cytokines and mediators are essential for host survival following infection and are also required for the repair of tissue injuries [11,12]. Over-expression of the inflammatory mediators in macrophage is involved in many inflammations related diseases, such as rheumatoid arthritis, atherosclerosis, chronic hepatitis and pulmonary fibrosis [13]. Thus, inhibition of these inflammatory mediators is an important strategy in the treatment of inflammatory diseases.

In mammalian cells, NO is synthesized by three different isoforms of nitric oxide synthase (NOS): endothelial NOS (eNOS), neuronal NOS (nNOS) and iNOS. Importantly, iNOS is highly expressed in LPS-activated macrophages and contributes to the pathogenesis of septic shock [14,15]. Cyclooxygenase (COX) is an enzyme that catalyzes the conversion of arachidonic acid to prostaglandin H2, a precursor for a variety of biologically active mediators, such as prostaglandin E2 (PGE_2_), prostacyclin and thromboxane A_2_ [16,17]. Two isoforms of COX have been found: COX-1 and COX-2. COX-2 is induced by several stimuli, and is responsible for the production of large amounts of pro-inflammatory prostaglandins at the inflammatory site [7,18]. PGE2 is derived from the catalyzation of arachidonic acid by COX-2 pathway. Therefore, NO production via iNOS pathway or PEG_2_ production via COX-2 pathway is the two key pathways in inflammatory processes.

In this study, as a part of our on-going screening program to evaluate the anti-inflammatory potential of marine-derived natural compounds, we investigated anti-inflammatory effects of broth and mycelium extracts of 16 marine fungi in RAW 264.7 cells. Furthermore, active compounds from the selected fungus were isolated and identified, and finally the anti-inflammatory effects and potential mechanisms of the isolated compounds were also evaluated in LPS-stimulated RAW264.7 cells.

## 2. Results

### 2.1. Culture and Isolation of Marine-Derived Fungi

A total of 16 fungi were isolated from four types of samples including algae, animals, floats and drift woods and then their strains were identified (Table 1). These fungal strains belong to four genera and two of them were unidentified. Most of the identified strains belong to the genus of *Penicillium* (7 strains), and three of them were from the genus of *Aspergillus.* The other species were of the genus of *Eurotium* and *Fusarium*. Although some of the isolated strains were the same, they were isolated from different hosts in the sea.

For evaluation of the biological activities of the extract from the fungal strains, all the strains were cultured in the flask with 100 mL of liquid culture medium. After several days, the fungal strains were further extracted to give broth extract (BE) and mycelium extract (ME) from broth and mycelium of the cultured strains. The BE and ME from each strain were checked on TLC to observe the profile of chemical composition (date not shown).

### 2.2. Cell Viability and NO Production Inhibitory Effects of the Extracts from Marine-Derived Fungi

**Figure 1:**
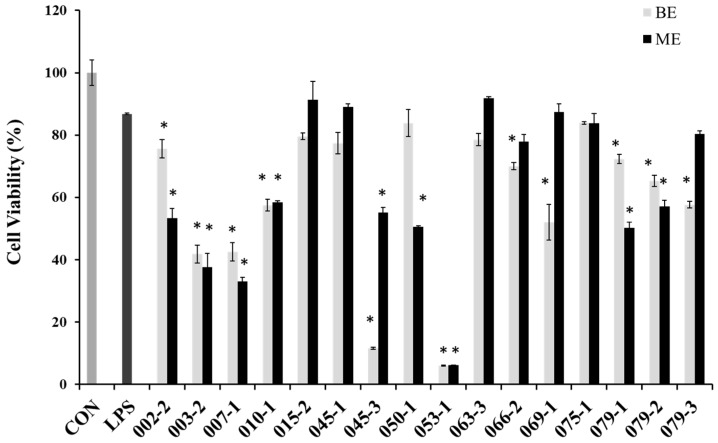
Cell viability of the extracts from marine-derived fungi in RAW 264.7 macrophages. Experiments were performed in triplicate and the data are expressed as mean ± SE. BE and ME represent the broth and mycelium extract obtained from each of the cultured fungus. * *p* < 0.05 indicates significant differences from the LPS-stimulated group.

In order to evaluate cytotoxicity of the extracts from marine-derived fungi in RAW 264.7 cells, cell viabilities were estimated via an MTT assay, which is a test of metabolic competence predicated upon the assessment of mitochondrial performance. As shown in Figure 1, the cells treated with LPS showed 87% cell viability, and some extracts showed strong cytotoxicity in RAW 264.7 cell. Especially, BE and ME of the unidentified strains of 003-2 and 007-1, as well as *P. chrysogenum* (010-1), *P. janthinellum* (053-1), *Penicillium* sp. (079-1), *P. chrysogenum* (079-2), *Penicillium* sp. (079-3) and the BE of *A. clavatus* (045-3), *F. oxysporum* (069-), the ME of *Fusarium* sp. (050-1), *P. crustosum* (066-2) showed lower cell viabilities than those of other extracts. However, the BE of *E. amstelodami* (015-2), *E. amstelodami* (045-1) and ME of *P. oxalicum* (075-1) showed slight cytotoxicities in RAW 264.7 cells. From the result it is very clearly proved that *E. amstelodami* is relatively non-toxic and can release non-toxic substances.

**Figure 2:**
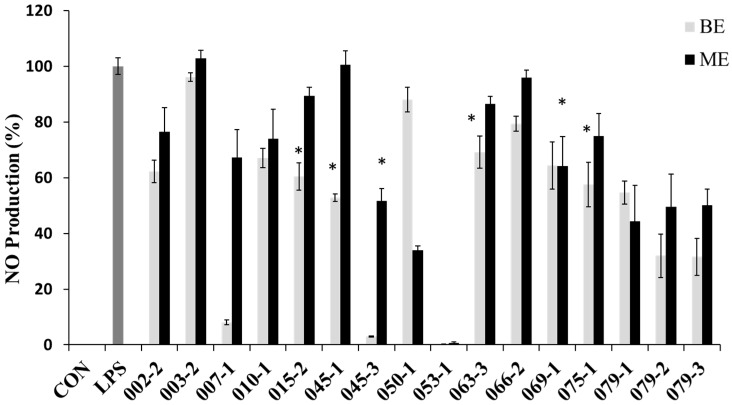
**Nitric oxide** (NO) production inhibitory effects of the extracts from marine-derived fungi. Each value indicates the mean ± S.D. and is representative of results obtained from three independent experiment. * *p* < 0.05 indicates significant differences from the LPS-stimulated group.

In order to evaluate the potential anti-inflammatory effects of the extracts with the protective effect against NO production in RAW 264.7 cells, the cells were treated with the extracts for 1 h and then treated with LPS (1 μg/mL) for 24 h. NO concentrations were measured in the culture supernatants by the Griess reaction and ELISA assay. The LPS treatment significantly increased the production of NO. However, the BE of *E. amstelodami* (015-2), *E. amstelodami* (045-1), *Aspergillus* sp. (063-3) and *P. oxalicum* (075-1) and ME of *A. clavatus* (045-3), *F. oxysporum* (069-1) showed significant inhibitory effects against NO production without cytotoxicity at concentration of 200 μg/mL. Especially, the BE of *E. amstelodami* (015-2), *E. amstelodami* (045-1), and *P. oxalicum* (075-1) showed the highest inhibitory activities for NO production with 60.4%, 52.9%, and 57.6% respectively (Figure 2). Therefore, these marine-derived fungi may produce anti-inflammatory substances.

### 2.3. Isolation and Identification of Bioactive Natural Products from E. Amstelodami

The strain, *E. amstelodami* (015-2) was selected for the further anti-inflammatory effects, therefore it was cultured for 30 d (static) at 29 °C in SWS medium. The broth and mycelium were separated by filtration. Its BE possessed higher NO production inhibitory activity than ME. Then, BE was purified using silica column chromatography followed by Sephadex LH-20 chromatography. Three compounds have been successfully isolated. The structures were identified by analyses of the MS and NMR data as well as comparison of those data with the previous reports. 

**Figure 3:**
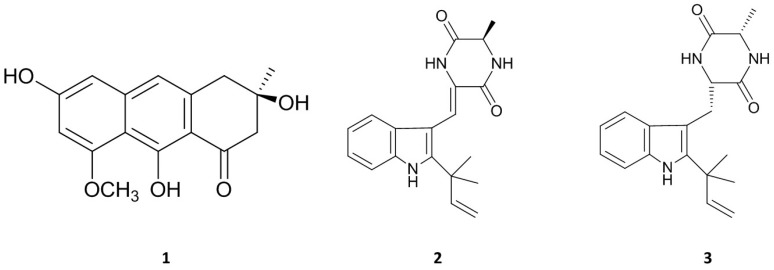
Chemical structures of three isolated compounds from marine-derived fungus, *E. amstelodami.*

Asperflavin (Figure 3 (**1**)): greenish amorphous powder, ESI-MS *m*/*z*: 287 [M − H]^−^. ^1^H-NMR (DMSO-*d*_6_, 400 MHz): δ 6.78 (1H, s, H-10), 6.54 (1H, d, *J* = 2.0, H-5), 6.42 (1H, d, *J* = 2.0, H-7), 3.84 (3H, s, CH3O-8), 2.95 (2H, s, H-4), 2.85 (1H, d, *J* = 16.9, H-2a), 2.79 (1H, d, *J* = 16.9, H-2b), 1.26 (3H, s, CH_3_-3). ^13^C NMR (DMSO-*d*_6_, 100 MHz): δ 203.2 (C-1), 165.0 (C-9), 161.2 (C-8), 160.6 (C-6), 141.6 (C-10a), 137.8 (C-4a), 115.7 (C-10), 108.8 (C-8a), 108.1 (C-9a), 101.9 (C-5), 97.8 (C-7), 69.4 (C-3), 55.7 (CH3O-8), 51.5 (C-2), 42.7 (C-4), 28.9 (CH3-3).

Asperflavin (Figure 3 (**1**)) was isolated as greenish amorphous powder. The LR-ESIMS data give a molecular ion peak at *m*/*z* 287 [M − H]^−^. The molecular formula of the compound was determined as C_16_H_16_O_5_ base on the MS and NMR data. The ^1^H and ^13^C-NMR data showed the presence of a carbonyl, a methoxyl, a methyl, two aliphatic methylenes, three aromatic protons among which two were meta-coupled with each other, an aliphatic quaternary carbon bearing oxygen, seven aromatic quaternary carbons among which three were bearing oxygen in the compound. It was determined as asperflavin as comparison with the previous report [19,20].

Neoechinulin A (Figure 3 (**2**)): white powder. ESI-MS *m*/*z*: 324 [M + H]^+^, ^1^H-NMR (DMSO-*d*_6_, 400 MHz): δ 1.38 (3H, d, *J* = 6.8, H-12), 1.47 (6H, s, CH_3_-18/19), 4.16 (1H, qd, *J* = 7.2, 1.6, H-12), 5.01 (1H, d, *J* = 13.1, Ha-17), 5.05 (1H, d, *J* = 6.2, Hb-17), 6.07 (1H, dd, *J* = 13.1, 6.2, H-16), 6.89 (1H, s, H-14), 7.01 (1H, dd, *J* = 7.6, 7.3, H-6), 7.08 (1H, dd, *J* = 7.8, 7.6, H-5), 7.19 (1H, d, *J* = 7.8, H-4), 7.42 (1H, d, *J* = 7.3, H-7), 8.32 (1H, s, H-8), ^13^C-NMR (DMSO-*d*_6_, 100 MHz): δ 166.4 (C-13), 159.9 (C-10), 145.1 (C-16), 143.9 (C-2), 135.1 (C-7a), 125.9 (C-3a), 124.9 (C-9), 120.7 (C-6), 119.3 (C-5), 118.8 (C-4), 111.6 (C-8), 111.5 (C-7), 110.1 (C-17), 103.4 (C-3), 50.5 (C-12), 38.9 (C-15), 27.5 (C-18/19), 19.6 (C-20).

Neoechinulin A (Figure 3 (**2**)), a white powder, has a molecular ion peak at *m*/*z* 322 [M − H]^−^. The molecular formula of the compound was determined as C_19_H_21_N_3_O_2_ base on the MS and NMR data. It was identified as neoechinulin A, which was previously isolated from fungal genera *Eurotium* and *Apsergillus* [21].

Preechinulin (Figure 3 (**3**)): white powder. ESI-MS *m*/*z*: 326 [M + H]^+^, ^1^H-NMR (DMSO-*d*_6_, 400 MHz): δ 1.23 (3H, d, *J* = 7.1, H-20), 1.49 (6H, s, CH_3_-18/19), 3.07 (1H, dd, *J* = 9.0, 14.4, Ha-8), 3.36 (1H, d, *J* = 4.6, Hb-8), 3.79 (1H, qd, *J* = 2.5, 7.0, H-12), 3.96 (1H, m, H-9), 5.04 (2H, d, *J* = 17.6, H-17), 6.18 (1H, dd, *J* = 10.5, 17.4, H-16), 6.93 (1H, dd, *J* = 7.3, 7.5, H-5), 7.02 (1H, dd, *J* = 7.7, 7.3, H-6), 7.31 (1H, d, *J* = 7.5, H-4), 7.42 (1H, d, *J* = 7.7, H-7), 7.49 (1H, d, *J* = 2.9, NH-14), 8.16 (1H, d, *J* = 2.5, NH-11), 10.5 (1H, s, NH-1). ^13^C-NMR (DMSO-*d*_6_, 100 MHz): δ 167.8 (C-13), 167.3 (C-10), 146.5 (C-16), 141.3 (C-2), 134.9 (C-7a), 128.9 (C-3a), 120.5 (C-6), 118.3 (C-5), 117.9 (C-4), 111.0 (C-17), 110.1 (C-7), 104.6 (C-3), 55.7 (C-9), 50.3 (C-12), 31.0 (C-8), 28.0 (C-19), 27.9 (C-18), 20.6 (C-20).

Preechinulin (Figure 3 (**3**)), a white powder, showed a molecular ion peak at *m*/*z* 326 [M + H]^+^. The molecular formula of the compound was determined as C_19_H_23_N_3_O_2_ base on the MS and NMR data. The structure of preechinulin was elucidated by analysis of its 1H, 13C-NMR and MS data, as well as by comparison of its spectral data to those reports in the literature [22].

### 2.4. Cell Viability of the Isolated Compounds in Raw 264.7 Cells

**Figure 4:**
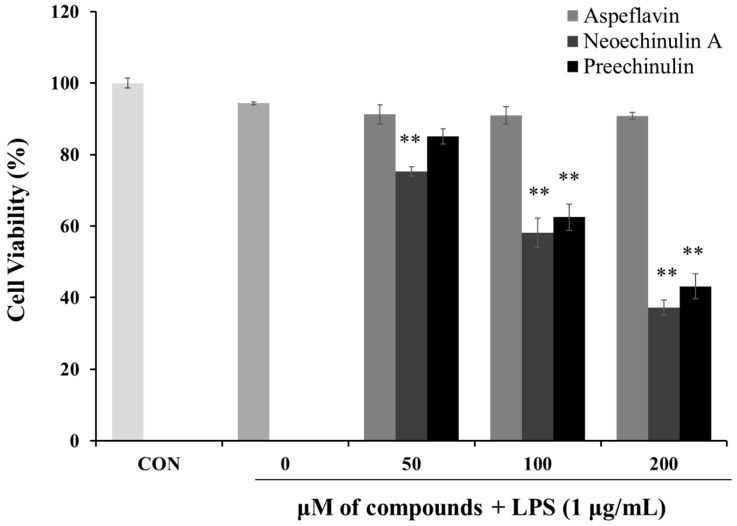
Cytotoxicities of asperflavin, neoechinulin A and preechinulin on cell viability in LPS-stimulated RAW 264.7 cells. RAW 264.7 cells were cultured with the different concentrations (50, 100, and 200 μM) of the compounds and LPS (1 μg/mL) for 24 h. Each value indicates the mean ± S.D. and is representative of results obtained from three independent experiment. ** *p* < 0.01 indicates significant differences from the LPS-stimulated group.

To investigate the cytotoxic effects of the isolated compounds in RAW 264.7 cells in the presence of LPS (1 μg/mL), MTT assays were employed. As shown in Figure 4, when the cells were treated with asperflavin at all the concentrations used in the study along with LPS, no significant differences in the cell viability were found, however, neoechinulin A and preechinulin indicated cytotoxic effects in RAW 264.7 cells. Thus, asperflavin was used for further experiments.

### 2.5. Effects of Asperflavin on NO and PGE_2_ Production in LPS-Treated RAW 264.7 Cells

**Figure 5:**
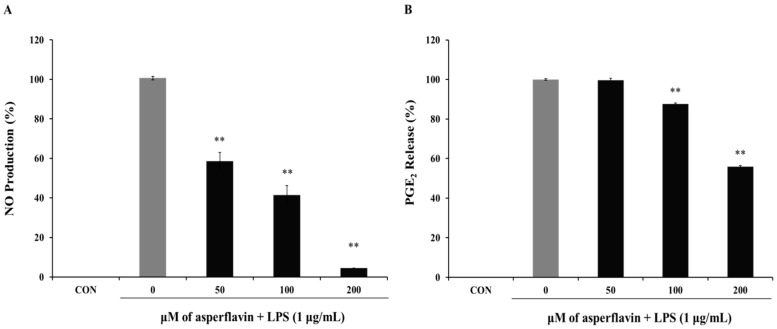
Inhibitory effect of asperflavin on NO (**A**) and PGE_2_ (**B**) production in LPS-stimulated RAW 264.7 cells. Cells (1 × 10^5^ cells/mL) were stimulated by LPS (1 μg/mL) for 24 h in the presence of asperflavin (50, 100, and 200 μM). The NO production was assayed in the culture medium and the PGE_2_ production in the supernatants was determined by ELISA. Each value indicates the mean ± S.D. and is representative of results obtained from three independent experiment. ** *p* < 0.01 indicates significant differences from the LPS-stimulated group.

The inhibitory effect of asperflavin on NO production in RAW 264.7 cells was evaluated and showed in the Figure 5A. Asperflavin could significantly inhibit LPS-induced NO production in a concentration-dependent manner and showed NO production of 58.5%, 41.4%, and 4.6% at the concentrations of 50, 100 and 200 μM, respectively.

The potential effects of asperflavin on the inhibition of PGE_2_ production were examined and shown in Figure 5B. Asperflavin could significantly inhibit LPS-induced PGE_2_ production in a concentration-dependent manner, except for the treatment at 50 μM of asperflavin. In the asperflavin-treated groups, the PGE_2_ production were 87.6%, and 55.9% at the concentrations of 100 and 200 μM, respectively.

### 2.6. Effects of Asperflavin on Expression of iNOS and COX-2 Protein in LPS-Stimulated RAW 264.7 Cells

**Figure 6:**
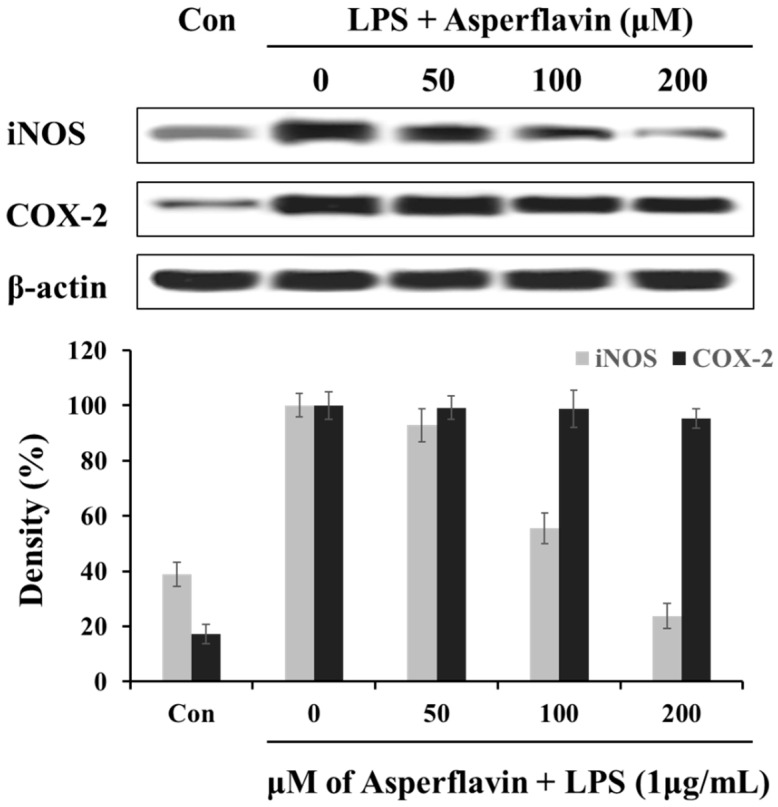
Inhibitory effects of asperflavin on the expression of iNOS and COX-2 in LPS-stimulated RAW 264.7 cells. The RAW 264.7 cells were pre-incubated for 18 h, and stimulated with LPS (1 μg/mL) for 24 h in the presence of asperflavin (50, 100, and 200 μM). The expression levels of iNOS and COX-2 were determined using immunoblotting method.

In order to understand the mechanism by which asperflavin reduces LPS-induced NO and PGE_2_ production, we investigated the ability of asperflavin on expression of iNOS and COX-2 in LPS-stimulated RAW 264.7 cells. As shown in Figure 6, the treatment of LPS (1 μg/mL) could significantly increase the expression levels of iNOS and COX-2 compared to the control without LPS, but asperflavin inhibited the expression of iNOS in a dose-dependent manner. On the other hand, the expression of COX-2 was not decreased by treatment of asperflavin at all the concentrations.

### 2.7. Inhibitory Effects of Asperflavin on Production of Pro-Inflammatory Cytokines in LPS-Stimulated RAW 264.7 Cells

**Figure 7:**
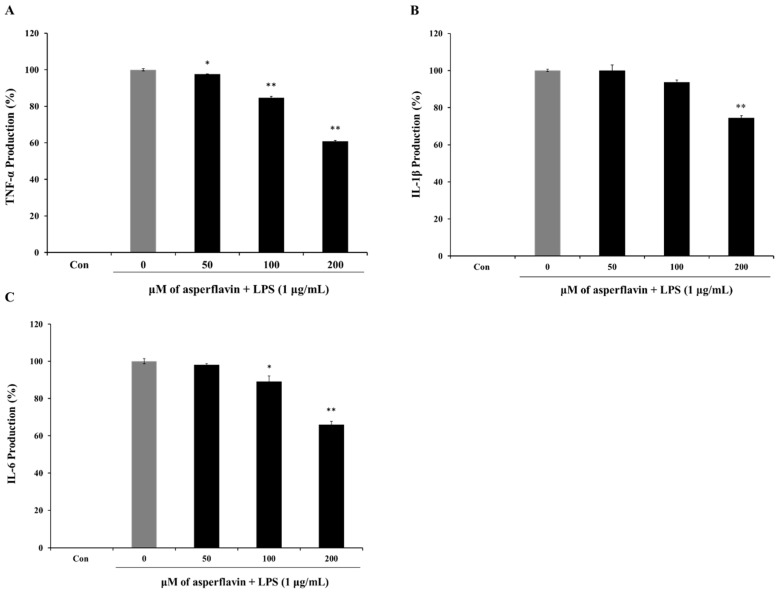
Inhibitory effects of asperflavin on TNF-α (**A**); IL-1β (**B**) and IL-6 (**C**) production in LPS-stimulated RAW 264.7 cells. The production of TNF-α was assayed in the culture medium of cells stimulated by LPS (1 μg/mL) for 24 h in the presence of asperflavin (50, 100, and 200 μM). Supernatants were collected, and the TNF-α, IL-1β and IL-6 production in the supernatants were determined by ELISA. Each value indicates the mean ± S.D. and is representative of results obtained from three independent experiment. * *p* < 0.05 and ** *p* < 0.01 indicate significant differences from the LPS-stimulated group.

The release of pro-inflammatory cytokines is an important mechanism by which the immune cells regulate the inflammatory responses and contribute to various inflammatory and autoimmune disorders. Therefore, we examined the effects of asperflavin on LPS-induced TNF-α, IL-1β, and IL-6 production using ELISA kit. LPS could induce a significant increase of cytokines including TNF-α (Figure 7A), IL-1β (Figure 7B) and IL-6 (Figure 7C) compared to the control group. However, pre-treatment with asperflavin significantly reduced the productions of all the pro-inflammatory cytokines at the concentrations of 200 μM. At the other lower concentrations of 50 and 100 μM, the levels of TNF-α were significantly reduced, but no significant reductions of IL-1βwere observed. The production of IL-6 in the asperflavin-treated group at the concentration of 50 μM was no significant different compared with LPS-treated group.

## 3. Discussion

Marine microorganisms are recognized as important sources of pharmacologically active metabolites [23]. In particular, marine-derived fungi have proven to be a promising source of structurally novel and biologically active secondary metabolites [24,25]. In this study, the 16 fungal strains have been isolated and belong to the genus of *Penicillium*, *Aspergillus*, *Eurotium*, *and Fusarium.* According to the results of cell viability and NO production of BE and ME from the marine fungi in LPS-treated RAW 264.7 cells, the BE of *Eurotium amstelodami* (015-2), *Eurotium amstelodami* (045-1), and *Penicillium oxalicum* (075-1) showed significant NO production inhibitory effects without cytotoxicity. Thus, the fungal strains could be good sources for biological natural products. In particular, *E. amstelodami* (015-2) was selected as our target strain for further studies. Previously, *E. amstelodami* was isolated from intermediate- or low-moisture food and in or near building air samples [26,27,28]. Slack also reported that the major secondary metabolites of *E. amstelodami* were neoechinulin A, neoechinulin B, epiheveadride, flavoglaucin, auroglaucin, and isotetrahydroauroglaucin [28]. Interestingly, this strain was found that it could growth well in the marine environment in our study.

Although natural products isolated from marine-derived fungi have been reported to possess various biological activity, the research focusing on their anti-inflammatory activity is few. Penstyrylpyrone, a new styrylpyrone-type metabolite isolated from marine-derived fungus *Penicillium* sp. was found to inhibit PTP1B activity and reduce NO, PGE_2_, TNF-α and IL-1β production through NF-κB pathway as well as expression of anti-inflammatory HO-1 [29]. Penicillinolide A, a new 10-membered lactone isolated from *Penicillium* sp., could suppressed the production of pro-inflammatory mediators via inhibition of the NF-κB pathway [30]. Shin reported a new tazawaic acid derivative, tanzawaic acid Q, together with four known compounds. The new compound was found to inhibit LPS-induced NO and PGE2 production via suppression of the expression of iNOS and COX-2 proteins in RAW264.7 cells [31].

As the result, three compounds, asperflavin, neochinulin A and preechinulin were successfully isolated from BE of *E. amstelodami*. Neoechinulin A has been demonstrated to have various biological activities, such as anti-tumor, anti-oxidant, and pro-inlfammatory activities. The study of Kim et al. indicated that neoechinulin A could suppress the production of pro-inflammatory mediators, and cytokines in LPS-induced RAW264.7 macrophages. The potential mechanism of its anti-inflammatory activity was due to the inhibition of NF-κB and p38 MAPK pathways [32]. However, in our study, neochinulin A and preechinulin has shown the significant cytotoxicity in RAW264.7 cells at the concentration of 50 to 200 μM. Asperflavin was initially isolated from entomogenous strain of *Aspergillus flavus*. It was also found in the marine-derived fungi of *Eurotium rubrum, Eurotium repens*, *Eurotium cristatum* and the fungus of *Eurotium herbariorum* which was used in the process of manufacturing karebushi (a katsuobushi) [33,34,35]. However, there are few reports to date on the biological activity of asperflavin. In this study, asperflavin indicated no-toxicity up to the concentration of 200 μM. Asperflavin significantly inhibited the LPS-induced NO and PGE_2_ production but the expression of iNOS was only significantly suppressed, not COX-2. Therefore asperflavin might go through the pathway of iNOS inhibition. Additionally, asperflavin significantly inhibited the production of pro-inflammatory cytokines, TNF-α, IL-1β and IL-6 in the LPS-stimulated RAW 264.7 macrophages.

## 4. Materials and Methods

### 4.1. Chemicals and Materials

Column chromatography were carried out by Silica Gel 60 (230–400 mesh, Merck, Darmstadt, Germany), ODS (12 nm, YMC, Kyoto, Japan), Sephadex LH-20 (Sigma, St. Louis, MO, USA). Thin-layer chromatography (TLC) was run on pre-coated Merck Kieselgel 60 F254 plates (0.25 mm). 3-(4,5-dimethylthiazol-2-yl)-2,5-diphenylte-trazolium bromide (MTT) and lipopolysaccharide (LPS) were purchased from Sigma Chemical Co. (St. Louis, MO, USA). Dulbecco’s modified Eagile’s medium (DMEM), fetal bovine serum (FBS), penicillin-streptomycin and trypsine-EDTA were purchased from Gibco/BRL (Grand Island, NY, USA). The enzyme-linked immunosorbent assay (ELISA) kit for IL-1β, IL-6, TNF-α and Prostaglandin E_2_ (PGE_2_) were purchased from R&D systems Inc (Minneapolis, MN, USA). Antibodies against iNOS and COX-2 were obtained from Calbiochem (La Jolla, CA, USA) and BD Biosciences Pharmingen (San Jose, CA, USA, respectively). All of the solvent and chemicals used in this study were of reagent grades from commercial sources.

### 4.2. Fungus Strain

There were 16 fungal strains isolated from the marine-derived samples including algae, float, animal and drift wood collected from the coast of Jeju Island, Korea. The isolated marine-derived fungi were identified according to a molecular biological protocol by DNA amplification and sequencing of ITS region (Table 1). The voucher specimens are deposited at Jeju National University.

For evaluation of the biological activities of the extracts from the fungal strains, all the strains were cultured in the flask with 100 mL of liquid culture medium. After several days, the cultured fungus was filtered through cheese-cloth to separate into broth and mycelium. The broth was extracted with EtOAc, and the EtOAc solution was concentrated under a reduced pressure to give a broth extract (BE). The mycelium was freeze-dried and extracted three times with CHCl_3_–MeOH (1:1) for 2 h using sonication and gave a mycelium extract (ME). The samples of BE and ME from each strain were checked by TLC to observe the profile of chemical composition (date not shown). All the extracts were prepared with different solvents for determination of cell viability and NO production in RAW264.7 cells.

### 4.3. Extraction and Isolation of Active Compounds from E. Amstelodami

According to the results of MTT and NO production assays, the strain, *E. amstelodami* (015-2) was selected as the target strain for the isolation of nature products and evaluation of their anti-inflammatory activity. The fugal strain was cultured (8 L) for 30 d (static) at 29 °C in SWS medium containing of soytone (0.1%), soluble starch (1.0%) and seawater (100%). The BE were prepared according to the above methods (015-2B) (0.7 g). The BE (0.7 g) which showed higher inhibitory effect against NO production was subjected to silica gel flash chromatography (*n*-hexane/EtOAc, EtOAc/MeOH) to furnish twelve fractions (Fr. B1-B12) on the basis of TLC analysis. Fr. B7 (161.2 mg) was further purified by Sephadex LH-20 column eluting with MeOH to give crude compound **1** and compound **2**. Final purification of each crude compound by HPLC (Sunfire, Waters, 50% MeOH) yielded the compound **1** (25.2 mg) and 2 (35.6 mg). Fr. B10 (130.5 mg) was further purified by Sephadex LH-20 column eluting with MeOH to give crude compound **3**. Then, the fraction was finally purified by ODS column to afford compound **3** (4.6 mg).

The ^1^H-NMR and ^13^C-NMR spectra of the isolated compounds were recorded on a JEOL JNM-ECP 400 MHz NMR spectrometer, using DMSO-*d*_6_ solvent peak (2.50 ppm in ^1^H and 39.5 ppm in ^13^C-NMR) as an internal reference standard. MS spectra were obtained on a JEOL JMS-700 spectrometer. 

### 4.4. Cell Culture

The murine macrophage cell line RAW 264.7 was purchased from the Korean Cell Line Bank (KCLB, Seoul, Korea). RAW 264.7 cells were cultured in DMEM supplemented with 100 U/mL of penicillin, 100 μg/mL of streptomycin and 10% FBS. The cells were incubated in an atmosphere of 5% CO_2_ at 37 °C and were sub-cultured every 3 days.

### 4.5. MTT Assay

MTT assay was adopted for evaluation of cytotoxicity. The RAW 264.7 cells were seeded in 96-well plate at a concentration of 5 × 10^5^ cells/ml (180 μL). After 24 h incubation at 37 °C under a humidified atmosphere, the cells were treated with 10 μL of the extracts of sixteen fungi (200 μg/mL) or the isolated compounds at the concentration of 50, 100 and 200 μM, and further incubated for 30 min. The 50 μL of MTT stock solution (2 mg/mL) was then applied to the wells, to a total reaction volume of 250 μL. After 24 h of incubation, the plates were centrifuged for 5 min at 800× *g*, and the supernatants were aspirated. The formazan crystals in each well were dissolved in 150 μL of dimethyl sulfoxide (DMSO), and the absorbance was measured via ELISA at a wavelength of 540 nm. The percentage inhibitory effect was evaluated in accordance with the quantity of MTT converted to the insoluble formazan salt. The optical density of the formazan generated in the control cells was considered to represent 100% viability. The data are expressed as mean percentages of the viable cells versus the respective control.

### 4.6. Determination of NO Production

RAW 264.7 cells (5 × 10^5^) were plated and incubated with the extracts (200 μg/mL) and the compounds at the concentrations of 50, 100 and 200 μM in the absence or presence of LPS (1 μg/mL) for 24 h. The nitrite concentration in the culture medium was measured as an indicator of NO production, according to the Griess reaction. One hundred microliters of the culture supernatant were mixed with the same volume of Griess reagent (0.1% naphtylethylenediamine dihydrochloride and 1% sulfanilamide in 5% H_3_PO_4_). The absorbance of the mixture was measured with a microplate reader (Ultraspec 2100 pro, Cambridge, UK) at 540 nm. The concentration of nitrite was calculated with sodium nitrite as a standard.

### 4.7. Determination of PGE_2_ Production

RAW 264.7 macrophages were pre-treated with 1 h with the active compound at the concentrations of 50, 100, 200 μM prior to 24 h of stimulation with LPS (1 μg/mL). The culture supernatants were immediately utilized for PGE_2_ determination. The PGE_2_ concentration in the culture medium was quantified using a competitive enzyme immunoassay kit according to the manufacturer’s instructions. The production of PGE_2_ was measured and indicated with comparing to the control treatment.

### 4.8. Western Blot Analysis

RAW 264.7 macrophages were pre-incubated for 24 h, and then stimulated with LPS (1 μg/mL) in the presence of the active compound for the indicated time. After incubation, the cells were collected and washed twice with cold-PBS. The cells were lysed in a lysis buffer [50 mM Tris-HCl (pH 7.5), 150 mM NaCl, 1% Nonidet P-40, 2 mM EDTA, 1 mM EGTA, 1 mM NaVO_3_, 10 mM NaF, 1 mM dithiothreitol, 1 mM phenylmethylsulfonylfluoride, 25 μg/mL aprotinin, 25 μg/mL leupeptin] and kept on ice for 30 min. Cell lysates were washed by centrifugation, and protein concentrations were determined by using BCA™ protein assay kit. Aliquots of the lysates (30–50 μg of protein) were separated on a 12% SDS-polyacrylamide gel and transferred onto a polyvinylidene fluoride (PVDF) membrane (BIO-RAD, HC, USA) with a glycine transfer buffer [192 mM glycine, 25 mM Tris-HCl (pH 8.8), 20% MeOH (*v*/*v*)]. After blocking the nonspecific site with 1% bovine serum albumin (BSA), the membrane was then incubated with specific primary antibody at 4 °C for overnight. The membrane was further incubated for 60 min with a peroxidase-conjugated secondary antibody (1:5000, Vector Laboratories, Burlingame, CA, USA) at room temperature. The immune-active proteins were detected using an enhanced chemiluminescence (ECL) western blotting detection kit.

### 4.9. Measurement of Pro-Inflammatory Cytokines Production

In order to determine the inhibitory effects of the active compound on production of pro-inflammatory cytokines including TNF-α, IL-1β and IL-6, the RAW 264.7 macrophages were incubated with the active compound (50, 100 and 200 μM) in the presence or absence of LPS (1 μg/mL) for 24 h. The inhibitory effects of the compound on the production of pro-inflammatory cytokines in LPS-treated RAW 264.7 cells was determined as described in the Cho et al protocols [36]. Supernatants were used for pro-inflammatory cytokines assay using mouse ELISA kit.

### 4.10. Statistical Analysis

All the data were presented as mean ± S.D. from three independent experiments unless stated otherwise. Statistical comparisons between different treatments were done by one-way ANOVA with Student Newman Keul’s post hoc tests using SPSS program (version 19.0). * *p* < 0.05, and ** *p* < 0.01, vs. LPS-stimulated group.

## 5. Conclusions 

In conclusion, we have demonstrated that asperflavin isolated from the marine-derived fungus, *E. amstelodami*, inhibited the production of NO and PGE_2_ production through suppression of iNOS expression in LPS-stimulated RAW 264.7 macrophages. Asperflavin was also found to inhibit the pro-inflammatory cytokines including TNF-α, IL-1β and IL-6 as well. Therefore, our study suggests that asperflavin might be considered a promising agent for the prevention and therapy of inflammatory disease.

## Figures and Tables

**Table 1:** Fungal strains isolated from marine resources.

Name	Host	Similarity (%)	No.
Unkown	Float		003-2
Unkown	Drift wood		007-1
*Aspergillus clavatus*	Drift wood	100	045-3
*Aspergillus tamarii*	Float	98	002-2
*Aspergillus* sp.	Alga	98	063-3
*Eurotium amstelodami*	Animal	100	015-2
*Eurotium amstelodami*	Float	100	045-1
*Fusarium oxysporum*	Alga	100	069-1
*Fusarium* sp.	Alga	100	050-1
*Penicillium chrysogenum*	Alga	100	010-1
*Penicillium chrysogenum*	Float	99	079-2
*Penicillium crustosum*	Alga	100	066-2
*Penicillium janthinellum*	Animal	96	053-1
*Penicillium oxalicum*	Alga	100	075-1
*Penicillium* sp.	Alga	99	079-1
*Penicillium* sp.	Alga	100	079-3
